# Quantification and Imaging of Antigens on Cell Surface with Lipid-Encapsulated Fluorescent Nanodiamonds

**DOI:** 10.3390/mi10050304

**Published:** 2019-05-06

**Authors:** Feng-Jen Hsieh, Yen-Wei Chen, Yuen Yung Hui, Chun-Hung Lin, Huan-Cheng Chang

**Affiliations:** 1 Institute of Atomic and Molecular Sciences, Academia Sinica, Taipei 106, Taiwan; fengjen.hsieh@gmail.com (F.-J.H.); s123428@yahoo.com.tw (Y.-W.C.); yyhui@pub.iams.sinica.edu.tw (Y.Y.H.); 2 Taiwan International Graduate Program—Chemical Biology and Molecular Biophysics, Academia Sinica, Taipei 115, Taiwan; chunhung@gate.sinica.edu.tw; 3 Institute of Biochemical Sciences, National Taiwan University, Taipei 106, Taiwan; 4 Institute of Biological Chemistry, Academia Sinica, Taipei 115, Taiwan; 5 Department of Chemical Engineering, National Taiwan University of Science and Technology, Taipei 106, Taiwan

**Keywords:** antigen, cell membrane, diamond nanoparticle, fluorescence microscopy, magnetic modulation

## Abstract

Quantifying the density and locating the position of antigens on cell surface has been a challenge in molecular biology research. The challenge lies in the need for a chemically and photophysically stable fluorophore to achieve the required sensitivity and accuracy. Here, we present a method suitable for the purpose by using lipid-encapsulated fluorescent nanodiamonds (FNDs) of 35 nm in diameter as biolabels. The encapsulation of FNDs in biotinylated phospholipids not only facilitates good dispersion of the particles in biological buffers, but also endows them with high specific targeting ability. We demonstrated a viable application of the technique for biotin-mediated immunostaining of antigens on fixed human cells, identifying their positions by two-color confocal fluorescence imaging, and determining their densities by magnetically modulated fluorescence detection. A binding capacity of 6 ± 1 × 10^4^ antigens/cell was measured specifically for CD44 on HeLa cell surface. The result agreed well with the assay of R-phycoerythrin-conjugated antibodies by flow cytometry, supporting the reliability of this new nanoparticle-based method.

## 1. Introduction

The surface of a cell is covered with various kinds of antigens [1]. These antigens are important molecular markers for the identification of different cell types and specific targets for the treatment of different diseases [2,3,4,5,6]. Red blood cells, for example, are classified as A, B, and O groups according to inherited differences in cell surface antigens composed of carbohydrates [4]. Human leukocyte antigens (or CD antigens), on the other hand, are proteins, and they play a central role in immune response [5]. The significance of these membrane-bound molecules has stimulated the development of an immunoenzymatic technique known as cell-enzyme-linked immunosorbent assay (cell-ELISA) to achieve quantitative analysis of cell surface antigens [7,8,9]. While the ELISA-based assay is highly sensitive, it does not provide information about the location of antigens on the cell surface. In contrast, atomic force microscopy [10] and optical microscopy [11,12,13,14] serve the latter purpose well, but are unable to determine the antigen densities with sufficient accuracy. Aiming to overcome these limitations, we have developed in this work a method that allows not only quantification of cell surface antigens, but also their spatial localization with nanometric resolution. Distinct from cell-ELISA, the technique involves no enzymes, radioactive materials, or antigen extraction. The key component of the technology is the lipid-encapsulated fluorescent nanodiamond (FND), which hosts a high-density ensemble of negatively charged nitrogen-vacancy (NV^–^) defects as fluorescent centers [15].

The NV^–^ centers in FNDs are atom-like light emitters, and thus are exceptionally photostable [16]. They are magneto-optical, and their fluorescence intensity can be modulated by applying an external magnetic field to alter the spin polarization of the centers at the ground electronic states [17]. Moreover, the NV^–^ emission has a relatively long decay time, up to 20 ns for particles dispersed in water and physiological media [18]. These characteristics together have enabled background-free imaging and fluorescence detection of FNDs in cell and tissue samples by using time gating and magnetic modulation techniques [19,20,21]. Another unique feature of the FND is that the NV^–^ centers are deeply embedded in the chemically inert diamond matrix, and, therefore, their fluorescence properties are largely unaffected by surface modifications and environmental changes at room temperature [18], which makes them useful as contrast agents for correlative light-electron microscopy (CLEM) [22,23], and well suited for quantitative applications even under extreme conditions [24]. It provides a robust new tool for both nanoscale localization and absolute quantification of antigens on cell surface, a task not achievable with conventional molecular fluorophores such as organic dyes and fluorescent proteins.

In a previous study, we developed a simple method to encapsulate FNDs in biotinylated lipid layers to overcome the hurdles of particle agglomeration and nonspecific interaction when using them as fluorescent biolabels [22,25]. It involves the addition of a mixture of phosphatidylcholine (PC), PEGylated 1,2-distearoyl-sn-glycero-3-phosphoethanolamine (PEG-DSPE), biotinylated PEG-DSPE, and cholesterol in tetrahydrofuran to an aqueous solution containing surface-oxidized FNDs to form emulsions. Subsequent evaporation of the tetrahydrofuran in a vacuum allows the lipid layer to encapsulate FNDs. The method has enabled robust coating as well as easy synthesis of biotinylated lipid-coated FNDs, which exhibit both high dispersibility in cell medium and high specific labeling and targeting abilities for cell surface antigens. A localization accuracy of ≈50 nm for CD44 antigens on the outer membrane of HeLa cells was achieved with lipid-encapsulated FNDs of ≈100 nm in diameter by CLEM [22]. This work represents an extension of that study by using FNDs in the size range of 35–100 nm for two-color confocal fluorescence imaging of cell antigens through biotin-mediated immunostaining, and to quantify cell antigen densities by magnetically modulated fluorescence (MMF) detection.

## 2. Materials and Methods 

Synthetic type Ib diamond powders (Micron + MDA) were obtained from Element Six; egg PC, cholesterol, 18:0 PEG2000 PE, and DSPE-PEG2000 biotin were from Avanti; Dulbecco’s modified Eagle’s medium (DMEM) and phosphate-buffered saline (PBS) were from Gibco; biotinylated anti-CD44 antibody was from Biolegend; NeutrAvidin and DyLight 488-conjugated NeutrAvidin were from Thermo Scientific; Atto542-biotin was from ATTO-TEC; paraformaldehyde was from Electron Microscopy Sciences; bovine serum albumin (BSA), tetrahydrofuran (THF), and all other chemicals were from Sigma-Aldrich and were used without further purification.

To prepare FNDs, diamond powders containing ≈100 ppm of atomically isolated nitrogen were radiation-damaged by ion bombardment to create carbon vacancies in the crystal lattice, followed by annealing in a vacuum at 800 °C to form NV^‒^ centers [26]. The as-produced FNDs were then oxidized in air at 450 °C for 1 h to remove graphitic carbon atoms on surface, and subsequently suspended in deionized distilled water (DDW) for conjugation with lipids. Particles of 35 and 50 nm in diameter were obtained by high-pressure crushing of 100 nm FNDs, separated by differential centrifugation, and characterized by dynamic light scattering (DLS). 

Biotinylated lipid-encapsulated FNDs (bL-FNDs) were prepared with a solvent evaporation method, as detailed elsewhere [22]. Briefly, egg PC, cholesterol, DSPE-PEG2000 biotin, and 18:0 PEG2000 PE were dissolved together in THF. The lipid mixture was then slowly dropped into the FND suspensions at 60 °C and immediately shaken for 30 s. After formation of a homogeneous lipid-FND-H_2_O solution, THF was removed by using a rotary evaporator to obtain bL-FNDs. To eliminate excess chemicals, the lipid-encapsulated particles were cleaned twice with DDW by centrifugal separation. Hydrodynamic diameters of the particles suspended in water before and after lipid encapsulation were measured by DLS with a particle size and zeta potential analyzer (Delsa Nano C, Beckman-Coulter, Brea, CA, USA). 

A human cervical cancer line (HeLa) and two human breast cancer cell lines (ASB145-1R and MCF7) were used to test the efficiency and specificity of the bL-FND labeling. The cells of interest were first incubated in DMEM containing 10% fetal calf serum and 1% penicillin at 37 °C with 5% CO_2_. They were then seeded in 35 mm dishes with a density of 1 × 10^5^ cells/well (70% confluence) one day before labeling. After being washed three times with PBS and fixed with 4% paraformaldehyde for 10 min, the cells were blocked with 3% BSA in PBS for 30 min and labeled with biotinylated anti-CD44 antibodies in 3% BSA-PBS to target CD44 antigens on the cell surface for another 30 min. The anti-CD44-labeled cells were then washed with 3% BSA-PBS to remove free antibodies and stained with DyLight 488-conjugated NeutrAvidin in 3% BSA-PBS. Following the same 30 min incubation and 3% BSA-PBS wash, the cells were finally stained with bL-FNDs (100 μg/mL) in 3% BSA-PBS for 30 min and thoroughly washed with 3% BSA-PBS prior to imaging with confocal fluorescence microscopy.

Fluorescence images were acquired with a laser-scanning confocal fluorescence microscope system (SP-8, Leica Camera AG, Wetzlar, Germany) equipped with a supercontinuum white-light laser operating at 488 nm and 561 nm for the excitation of DyLight 488 and FND, respectively. The corresponding fluorescence was collected at 500–550 nm (DyLight 488) and 600–800 nm (FND) through an oil-immersion objective (63×, NA 1.4, Leica Camera AG, Wetzlar, Germany). 

Flow cytometry was carried out using a cytometer (FACSArray Bioanalyzer, BD Bioscience) equipped with a 532 nm laser as the light source. To conduct the analysis, cells were first trypsinized and divided into 15 mL tubes (TPP Techno Plastic Products AG, Trasadingen, Switzerland) with a cell number of 2 × 10^5^ per tube. The cells were then washed once with PBS and treated with 3% BSA in PBS for 30 min. After removal of the blocking buffer by centrifugation, cells were re-suspended and stained with biotinylated anti-CD44 antibodies. Incubated for another 30 min, the antibody-labeled cells were washed with 3% BSA-PBS three times to remove un-bonded antibodies, and NeutrAvidin was added to target biotinylated antibodies on cell surface. Following the same 30 min incubation and washes with 3% BSA-PBS, the cells were stained with 100 µg/mL bL-FND or 0.15 µg/mL ATTO542-biotin in 3% BSA-PBS for 0.5–2 h. Washes with 3% BSA-PBS were later performed three times to remove non-specific targeting of CD44 antigens by bL-FND. The cells (≈100,000) from each sample were finally analyzed by flow cytometry, with the fluorescence detected at > 590 nm.

The densities of antigens on the cells were determined by measuring the fluorescence intensities of FNDs using a home-built spectrometer, as described previously [24]. The spectrometer was equipped with a continuous-wave 532 nm laser (DPGL-2100F, Photop Suwtech, Hertfordshire, UK), a dichroic beam splitter (Z532RDC, Chroma, Bellows Falls, VT, USA), a long-working distance microscope objective (50×, NA 0.55, Mitutoyo, Kanagawa, Japan), a long-pass edge filter (FF01-732/68, Semrock, Rochester, NY, USA), and a multichannel analyzer (C7473, Hamamatsu, Hamamatsu, Japan). Backward fluorescence was collected through the same objective to minimize the effect of light scattering from FNDs. A calibration curve was first prepared by measuring the total intensities of the NV^–^ fluorescence at wavelengths longer than 600 nm as a function of FND concentration (1‒20 μg/mL). The fluorescence intensities of bL-FND-labeled cells (typically 5 × 10^5^ cells/mL) were then obtained after sonication of the cells in water for 1 h to break up cell membranes and release the particles into solution. A sinusoidal magnetic field was applied to the suspensions to selectively detect the fluorescence of FNDs by removing background signals derived mainly from the Raman scattering of water. For comparison purposes, the binding capacities of CD44 antigens on HeLa cells were independently quantified by using the QuantiBRITE-PE kit (BD Bioscience, San Jose, CA, USA), following the manufacturer’s instructions.

Transmission electron microscopy (TEM) of bare FNDs and bL-FNDs was carried out using a transmission electron microscope (JEM-1400, JEOL, Tokyo, Japan) at an energy of 120 keV. The samples were fixed on a copper grid, stained with saturated uranyl acetate for 1 min, and stored in a dry box for two days before use.

## 3. Results and Discussion

### 3.1. Flow Cytometry and Confocal Fluorescence Imaging of Cell Surface Antigens

**Figure 1:**
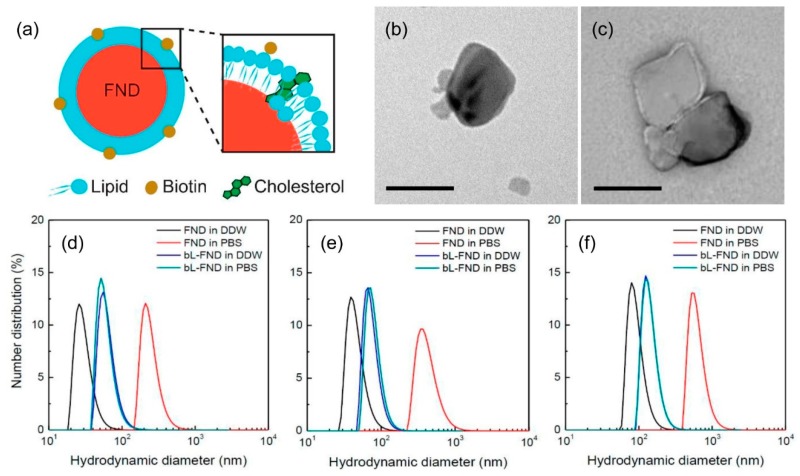
(**a**) Proposed structure of a biotinylated lipid-encapsulated (FND) (bL-FND). The structure is applicable for particles of different sizes, from 35 to 100 nm. (**b**,**c**) Transmission electron microscopy (TEM) images of bare FNDs (b) and bL-FNDs (c). Scale bars: 50 nm. (**d**–**f**) Number-averaged size distributions of FNDs with nominal diameters of 35 nm (d), 50 nm (e), and 100 nm (f) before and after coating with lipids in deionized distilled water (DDW) or phosphate-buffered saline (PBS), measured by dynamic light scattering (DLS).

BL-FNDs of three different sizes (with nominal diameters of 35, 50, and 100 nm) were synthesized and employed as biolabels in this work. Figure 1a shows a proposed structure of the bL-FND particle. A comparative study with TEM for bare FNDs (Figure 1b) and bL-FNDs (Figure 1c) revealed a thin layer of lipids formed on the surface after synthesis. The result was consistent with the measurement by DLS, showing a layer thickness of ≈10 nm for all three samples (Figure 1d–f). The typical content of DSPE-PEG2000 biotin in the lipid layer was 1% in molar ratio, which gave good dispersibility and high colloidal stability of the particles in both DDW and PBS [22]. 

**Figure 2:**
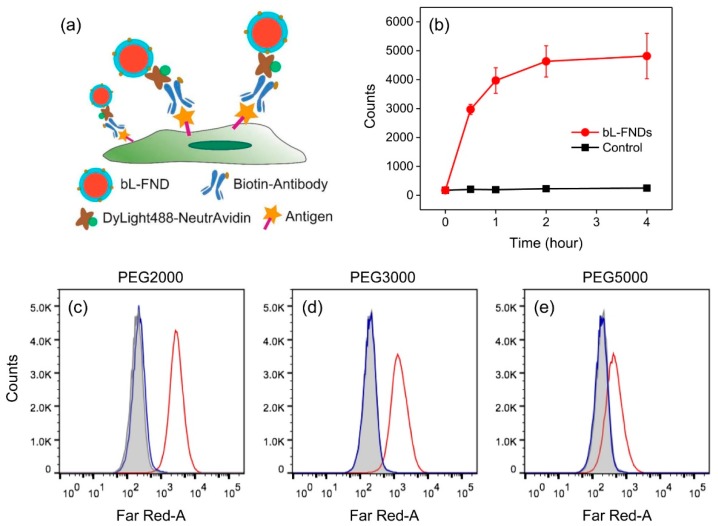
(**a**) Sandwich immunostaining of HeLa cells with bL-FNDs. (**b**) Flow cytometric analysis of fixed HeLa cells labeled with 100 nm bL-FNDs at various incubation times. Control experiments were performed following the same labeling procedures as in (a) but without NeutrAvidin. Error bars represent standard deviations of the measurements in triplicate. (**c**–**e**) Effects of PEG length on the labeling efficiency of bL-FNDs for HeLa cells: PEG2000 (c), PEG3000 (d), and PEG5000 (e). The bL-FND concentrations used in the labeling were all 100 μg/mL. Control experiments were performed with HeLa cells only (grey areas), or following the same labeling procedures but without NeutrAvidin (blue curves).

To optimize the targeting efficiency and specificity, a series of experimental conditions were evaluated by labeling CD44 antigens on the surface of fixed HeLa cells with 100 nm bL-FNDs through three step sandwich immunostaining (Figure 2a). This was accomplished by sequentially staining the cells with biotinylated anti-CD44 antibodies, NeutrAvidin, and bL-FNDs at a particle concentration of 100 μg/mL prior to flow cytometric analysis. As shown in Figure 2b, the efficiency of the labeling increased with increasing incubation time, but reached a plateau at ≈2 h. No significant non-specific interaction occurred over an extended period of the labeling. We next explored the effect of PEG length on the labeling capability of the particles. While increasing the PEG length helped reduce the degree of particle aggregation, it decreased the labeling efficiency, as revealed by flow cytometric analysis of HeLa cells incubated with bL-FNDs consisting of PEG2000, PEG3000, and PEG5000 in the lipid layers (Figure 2c–e). We therefore adopted PEG2000 as the anticoagulant and 2 h as the labeling time throughout the experiments.

**Figure 3:**
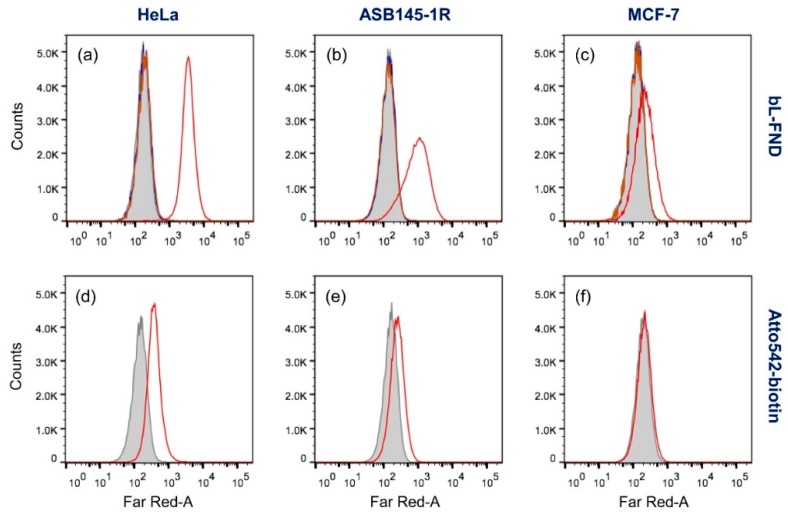
Flow cytometric analysis of HeLa (**a**,**d**), ASB145-1R (**b**,**e**), and MCF-7 (**c**,**f**) cells labeled with biotin-anti-CD44 antibody, NeutrAvidin, and bL-FND (a–c) or Atto542-biotin (d–f), respectively. Control experiments were performed following the same labeling procedures, but without NeutrAvidin (blue) or biotin-anti-CD44 antibody (orange). Cell-only results are given in grey.

In addition to HeLa cells, we also applied the FND-based immunostaining technique to other cell types (MCF-7 and ASB145-1R) and compared its performance with that of dye labeling (such as Atto542-biotin). MCF-7 and ASB145-1R are breast cancer cell lines with different levels of CD44 expressed on the cell surface, which is detectable by flow cytometry [27]. As shown in Figure 3a–c,d–f, good agreement between flow cytometric analysis of FND- and Atto542-labeled cells was reached for all three cell lines. The agreement between these two sets of data strongly suggests that the 100 nm bL-FNDs are able to specifically target CD44 antigens on human cell surface, and are thus useful as immunolabeling agents. Compared with the dye labeling using Atto542-biotin, the bL-FND labeling produced significantly stronger fluorescence signals, although they were much larger in size. It is noteworthy here that the molar concentration of the 100 nm bL-FND particles used in these labeling experiments was ≈0.1 nM (or ≈6 × 10^10^ particles/mL), given a weight concentration of 100 μg/mL and a weight of ≈2 fg per particle. This molar concentration is lower than that used (typically 0.1 µM) of Atto542-biotin by about 3 orders of magnitude. The outperformance of 100 nm bL-FND over Atto542-biotin is attributed to the high fluorescence brightness of the nanometer-sized particles, each of which contains roughly 900 NV^–^ centers in the diamond matrix [26].

**Figure 4:**
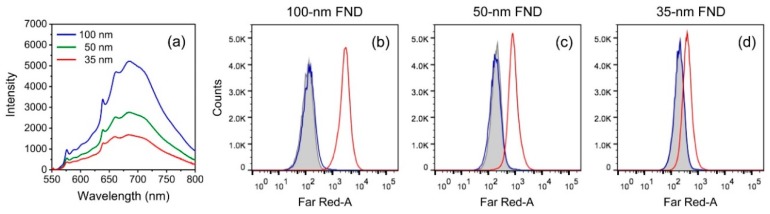
(**a**) Fluorescence spectra of FNDs suspended in water (100 μg/mL). The fluorescence intensities decreased with particle size from 100 nm (blue), to 50 nm (green), to 35 nm (red). (**b**–**d**) Flow cytometric analysis of HeLa cells labeled with (b) 100 nm, (c) 50 nm, and (d) 35 nm bL-FNDs. Control experiments were performed following the same labeling procedures, but without NeutrAvidin (blue) or biotin-anti-CD44 antibody (red). Cell-only results are in grey.

We further examined the effect of particle size on the cell labeling efficiency by flow cytometry. Our previous studies have found that the labeling of CD44 antigens on HeLa cell surface with 100 nm bL-FNDs was saturated at a particle concentration of 700 μg/mL [22], corresponding to ≈4 × 10^11^ particles/mL. This density is too low to achieve complete labeling. To overcome this limitation, we reduced the particle size from 100 nm to 50 and 35 nm, which effectively increased the particle number by 8- and 24-fold if the weight concentration was kept the same. The method significantly enhanced the labeling efficiency, but at the expense of signal strength. Figure 4a shows the fluorescence intensity of FNDs dispersed in water with particle sizes of 100, 50, and 35 nm. As can be seen, a reduction of the particle size from 100 to 50 nm resulted in a 2-fold decrease in the total fluorescence intensity for samples of the same weight concentration (100 μg/mL). This means that the fluorescence intensity of each 50 nm FND particle was about 16-fold weaker than that of the 100 nm one. The corresponding intensity changes were ≈3-fold and 80-fold, respectively, for 35 nm FNDs. The decrease of the total intensity is most likely due to the surface effect, which partially quenches fluorescence as particles become smaller. Although the effect is substantial, it does not prohibit the use of these smaller FND particles as biolabels. The results of the flow cytometric analysis of HeLa cells labeled with 100, 50, and 35 nm bL-FNDs in Figure 4b–d demonstrated the feasibility of this approach for practical applications.

**Figure 5:**
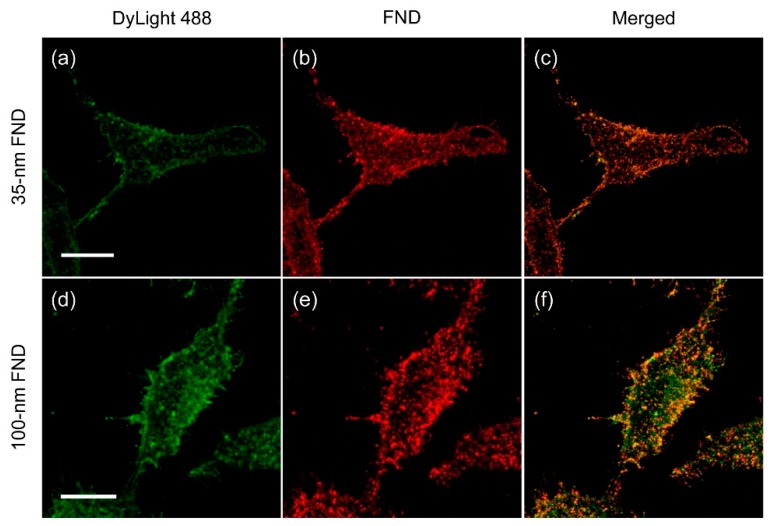
Co-localization studies with HeLa cells labeled with (**a**–**c**) 35 nm or (**d**–**f**) 100 nm bL-FNDs through sandwich immunostaining. The cells were first fixed and sequentially labeled with biotin-antiCD44 antibody, DyLight 488-NeutrAvidin (green), and bL-FNDs (red). Yellow spots in (c,f) indicate the co-localization of DyLight 488-NeutrAvidin (green) and bL-FNDs (red). The concentration of bL-FNDs used for the labeling was 100 μg/mL. Scale bars: 20 μm.

While using smaller FND particles is disadvantageous in having weaker fluorescence signals, more homogeneous labeling of antigens on cell surface was expected. The expectation was confirmed by using two-color confocal fluorescence imaging. In this experiment, HeLa cells were sequentially stained after formaldehyde fixation with biotinylated anti-CD44 antibodies, DyLight 488-conjugated NeutrAvidin, and bL-FNDs at a particle concentration of 100 μg/mL. Fluorescence images were then acquired by laser excitation at 488 nm for DyLight 488 and at 561 nm for FND, and their corresponding fluorescence emissions were collected at 500–550 nm and 600–800 nm, respectively. As shown in Figure 5a–c, good co-localization of the fluorescence signals of FND and DyLight 488-conjugated NeutrAvidin on the cells’ surface was achieved with the use of 35 nm bL-FNDs in the labeling. By comparison, many untargeted sites were found when 100 nm bL-FNDs were applied for the same purpose (Figure 5d–f), indicating incomplete labeling. The result highlights the importance of using 35 nm or smaller FNDs as biolabels.

### 3.2. Quantification of Cell Surface Antigens by Magnetic Modulated Fluorescence

The high labeling fidelity of 35 nm bL-FNDs signifies the potential use of the particles for the quantification of antigen densities on cell surface. Such information cannot be deduced solely from the confocal fluorescence images as shown in Figure 5. It is known that flow cytometry provides only a relative measure for the degree of cell labeling and, as a result, the determination of antigen density requires calibration against the molecules of equivalent fluorochromes, such as R-phycoerythrin, as used in the commercial QuantiBRITE-PE kit [28,29]. The ultimate sensitivity of this method is limited by cell autofluorescence backgrounds (Figure 4b–d). Such a limitation, however, is not an issue for FNDs, because one can easily perform magnetic modulation to remove background signals while detecting the fluorescence from NV^–^ centers. This allows absolute and sensitive quantification of antigen densities on the surface of bL-FND-labeled cells by fluorescence intensity measurements.

For the sake of comparison, we performed absolute quantification of bL-FNDs on the HeLa cell surface by MMF for particles of three different sizes under the same labeling conditions. Specifically, to prepare a homogeneous solution for fluorescence intensity measurements, cells labeled with bL-FNDs were first sonicated to release the particles from their membrane, and the fluorescence intensities of the resulting FND suspensions were then measured directly without pre-separation by using a home-built fluorescence spectrometer, featuring a magnetic modulation function to eliminate background noises. The fluorescence intensities thus obtained after fast Fourier transform (FFT) of the measured signals were finally compared against a calibration curve to extract the particle weight concentration, from which the numbers of particles per cell were estimated, assuming a spherical shape for the FNDs. The magnetic modulation function is especially crucial for measurement at FND concentrations of less than 1 μg/mL, where the Raman scattering of water dominates the observed spectra [24].

With the use of 100 nm bL-FNDs as the biolabels, we obtained an average value of 6 ± 1 × 10^3^ particles on each HeLa cell surface, which may represent only a fraction of the CD44 antigens on the membrane. Next, we conducted labeling with 50 nm bL-FNDs at the same weight concentration of 100 μg/mL. The number of the antigens detected significantly increased to 3 ± 1 × 10^4^, which further climbed up to 6 ± 1 × 10^4^ when 35 nm bL-FNDs were used for labeling. The trend of this change is in line with the relative number (i.e., 1:8:24) of these three types of particles in the cell medium, suggesting that the labeling of these cell antigens with bL-FNDs is not a diffusion-limited process, but is a particle-number-dependent event.

To verify the reliability of the absolute quantification method for cell surface antigens with 35 nm bL-FNDs, it was necessary to compare the obtained result with existing assays. For this purpose, we employed the QuantiBRITE-PE kit, which has been developed to determine the number of R-phycoerythrin (R-PE)-conjugated antibodies bound to a cell by flow cytometry, using amino-functionalized poly(methyl methacrylate) (PMMA) beads with a known number of attached R-PE molecules as the references [28,29]. R-PE was chosen for the quantitation of fluorochromes because the molecule lacks self-quenching, and can form well-defined antibody conjugates. Similar to the FND-based quantification, the antibody-binding capacity (ABC) of the cells was determined by comparing the measured fluorescence signals against a calibration curve prepared with the R-PE-conjugated PMMA beads (6 µm in diameter). With the use of this commercially available kit, we obtained a value of ABC = 6.2 × 10^4^, which matched closely with our measurement of 6 × 10^4^ using the 35 nm bL-FND particles. The agreement strongly implies that the labeling of CD44 antigens on the surface of HeLa cells with 35 nm bL-FNDs is monovalent. Interestingly, and also importantly, most of the epitopes in the antigens were still accessible by antibodies attached to the bL-FND nanobeads. The result, along with the fluorescence images in Figure 5a–c, lends support to the suggestion that 35 nm FNDs, after proper surface modification, are potentially useful as a tool to determine the ABC of cells without the need of fluorescent proteins like R-PE. The reliability of this assay can be further enhanced if monodispersed FNDs of smaller size (such as the 20 nm ones) are accessible and used for quantification.

## 4. Conclusions and Future Work

FND is a biocompatible nanoprobe with unique magneto-optical properties, including exceptionally high photostability, magnetically modulable fluorescence, and long-lived fluorescence decay. These properties together make it possible to achieve high-quality, background-free imaging and localization of cellular components with nanoscale resolution if the nanoparticles are endowed with specific targeting abilities. This work demonstrated their feasibility by using bL-FNDs to determine the density of CD44 antigens on HeLa cell surface by MMF detection. In particular, the result obtained with 35 nm bL-FNDs was in satisfactory agreement with the assays performed by using R-phycoerythrin-conjugated antibodies and flow cytometry. 

One of our future experiments will be focused on super-resolution imaging of cell surface antigens with stimulated emission depletion (STED) microscopy, which has achieved a resolution down to 20 nm for neurotrophin receptors using immunostaining and Huygens deconvolution [30]. Such a spatial resolution can be readily improved to 10 nm with STED [31] and STED-TEM [23] if FNDs smaller than 10 nm are available for labeling. With further improvement in fluorescence brightness and size uniformity, the lipid-encapsulated FNDs are expected to find broad and cutting-edge applications in life science research.
